# The management of women with thoracic endometriosis: a national survey of British gynaecological endoscopists

**Published:** 2021-01-08

**Authors:** M Hirsch, L Berg, I Gamaleldin, S Vyas, A Vashisht

**Affiliations:** Elizabeth Garrett Anderson Institute for Women’s Health, University College London, United Kingdom; Women’s Health, Southmead Hospital, Bristol, United Kingdom

**Keywords:** Endometriosis, thoracic endometriosis, diaphragmatic endometriosis, catamenial pneumothorax, catamenial haemothorax

## Abstract

**Objectives:**

This study evaluates current national opinions on screening, diagnosis, and management of thoracic endometriosis.

**Background:**

Thoracic endometriosis is a rare but serious condition with four main clinical presentations: pneumothorax, haemoptysis, haemothorax, and pulmonary nodules. There are no specialist centres in the United Kingdom despite growing patient desire for recognition, investigation, and treatment.

**Methods:**

We distributed a multiple-choice email survey to senior members of the British Society for Gynaecological Endoscopy. Descriptive statistics were used to present the results. Results: We received 67 responses from experienced clinicians having provided over 800 combined years of endometriosis patient care. The majority of respondents managed over 100 endometriosis patients annually, for more than five years. Over one third had never managed a patient with symptomatic thoracic endometriosis; just 9% had managed more than 30 cases over the course of their career. Screening varied by modality with only 4% of clinicians always taking a history of respiratory symptoms while 69% would always screen for diaphragmatic endometriosis during laparoscopy. The management of symptomatic thoracic endometriosis varied widely with the commonest treatment being surgery followed by hormonal therapies. Regarding management, 71% of respondents felt the team should comprise of four or more different specialists, and 56% believed care should be centralised either regionally or nationally.

**Conclusions:**

Thoracic endometriosis is poorly screened for amongst clinicians with varied management lacking a common diagnostic or therapeutic pathway in the United Kingdom. Specialists expressed a preference for women to be managed in a large multidisciplinary team setting at a regional or national level.

## Introduction

Thoracic endometriosis describes the presence of endometrial glands or stroma on the lung parenchyma, pleural surface or diaphragm (Andres et al., 2020). It has four main clinical presentations: catamenial pneumothorax (80%), catamenial haemothorax (14%), catamenial haemoptysis (5%) and pulmonary nodules (1%) (Alifano et al., 2006). Thoracic endometriosis is thought to be rare, but it is also a wholly underdiagnosed problem, and thus the true prevalence and age incidence are unknown, with little research existing beyond case reports. Indeed, recent hypothesis suggests that thoracic endometriosis may be more common than previously thought underlying 13% of idiopathic pneumothorax and 64% of catamenial pneumothorax (Alifano et al., 2007).

The aetiology of thoracic endometriosis is currently poorly understood with limited high-quality research evaluating diagnosis and treatment. Interestingly, thoracic endometriosis is right sided in 85% of cases. This may provide clues for its origins in the future. Optimal methods for diagnosis are not fully agreed. There is currently no national guidance on the investigation and management of women with thoracic endometriosis in the United Kingdom (UK). Perhaps as a reflection of this, as well as successful national campaigns to raise awareness of endometriosis by groups such as Endometriosis UK, there has been increased patient interest and request for greater recognition and expert treatment for sufferers.

The British Society for Gynaecological Endoscopy (BSGE) has established specialist endometriosis centres since 2007. This progressive approach to service delivery ensures centres meet pre-specified criteria to deliver specialist care to women with severe endometriosis. These include: a minimum number of procedures per annum per surgeon, a dedicated nurse specialist, a named urological and colorectal colleague for surgical support, and an annual exemplar video evaluation. This rigorous governance structure ensures standards of clinical and surgical care are maintained through regular evaluation. Despite this, there remains variation in access to treatment identified by women with thoracic endometriosis. A call for help from patients who have been successfully diagnosed with thoracic endometriosis in the UK was sent to the BSGE highlighting the conflicting advice and management received at the BSGE treatment centres and aimed to raise awareness of this debilitating condition.

Thoracic endometriosis, like pelvic endometriosis, poses significant diagnostic and therapeutic challenges, with delays commonly experienced. (Joseph and Sahn, 1996) This study aims to evaluate current practice in the diagnosis and management of thoracic endometriosis in the UK and to seek opinion amongst gynaecologists on how care should be optimally delivered for these patients. This is vital to provide an overview of how care is being provided and to inform the development of a diagnostic and therapeutic pathway for current and future patients with suspected thoracic endometriosis.

## Methods

This was a cross-sectional survey electronically distributed amongst members of the largest national organisation of endometriosis specialists in the UK. The target population was senior gynaecologists who provide specialist care to endometriosis patients. This was achieved through distribution to members of the BSGE.

The questionnaire was designed by all authors with pilot testing prior to circulation. All authors had prior experience of qualitative research and at the time of distribution authors SV and AV were senior council members of the BSGE. Invited participants were informed of the researcher’s names but not qualifications or demographic details.

The questionnaire was designed to assess screening, diagnosis, management, and service provision using content analysis. The major research themes were highlighted in advance (screening, diagnosis, management, and service provision) and formed the basis for the closed ended questions. The findings will be reported in line with a standardised criteria (Tong et al., 2007)

The survey comprised of 16 items assessing each clinician’s experience of managing patients with thoracic endometriosis and their opinion on how such care should optimally be provided (Figure 1). The survey was sent via email to all members of the BSGE. Responses from individuals were collated on Microsoft Excel spreadsheets on a password protected computer. Descriptive statistics were used to present results. Ethical approval was not sought as it was survey of clinical practice amongst healthcare professionals.

**Figure 1:**
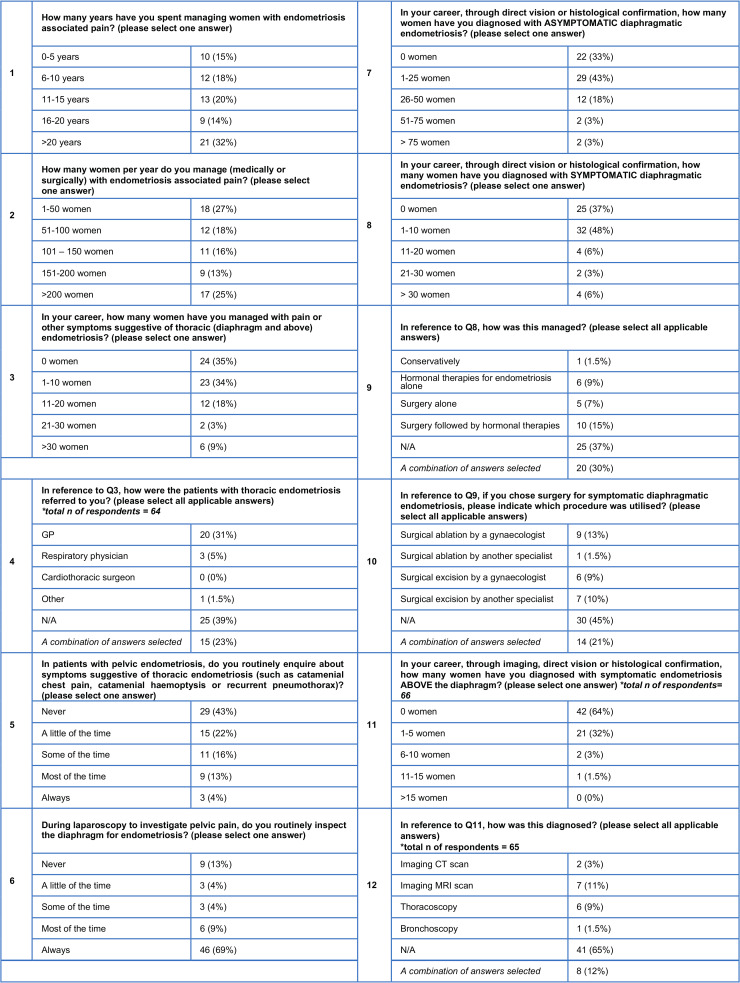
Part 1 - Questionnaire responses (total number of responses = 67).

**Figure 1:**
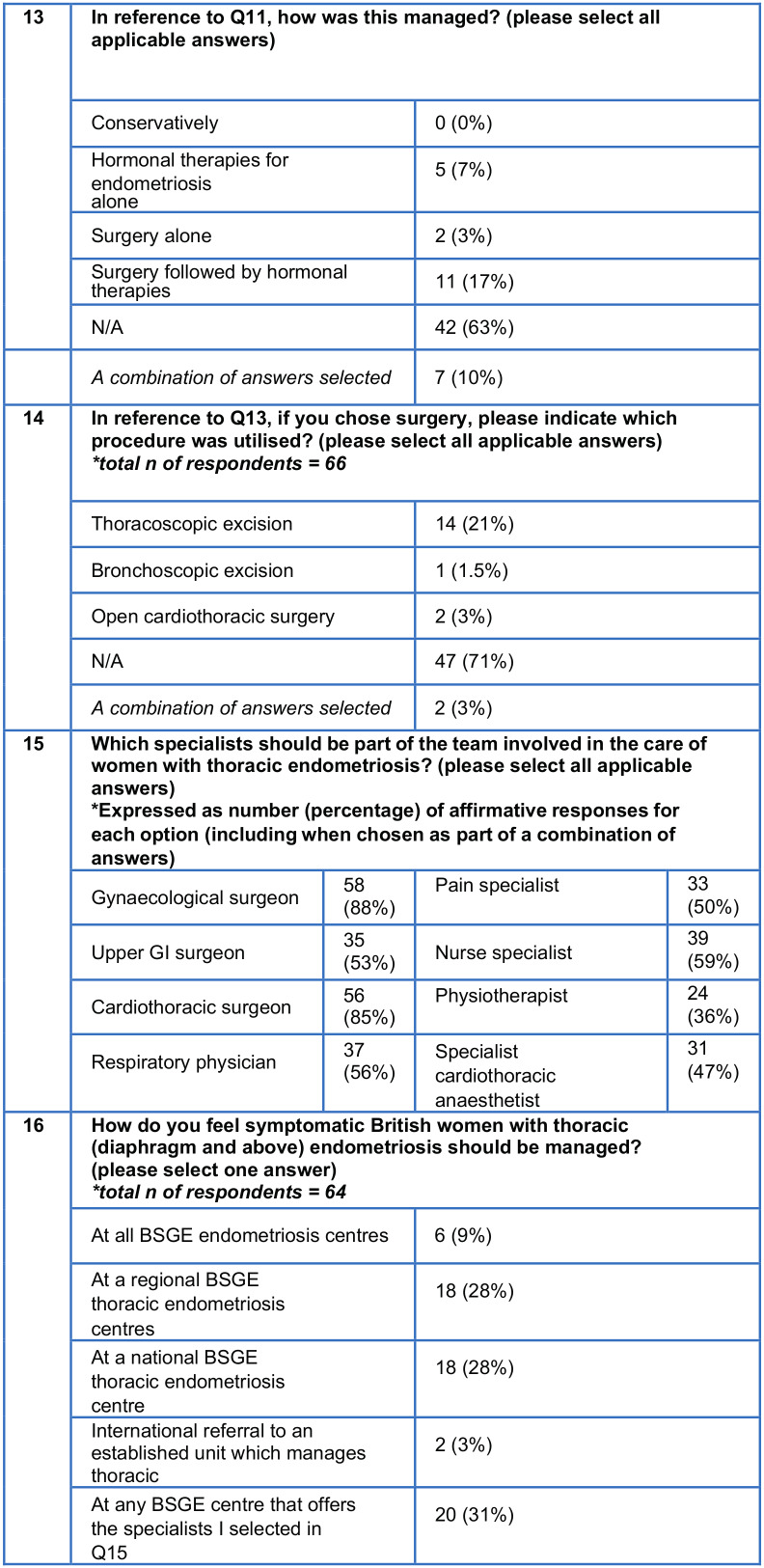
Part 2 - Questionnaire responses (total number of responses = 67).

To gauge service requirement, we sought to estimate the minimum and maximum number of cases of thoracic endometriosis requiring intervention per respondent per year in our survey. To estimate the minimum number of cases per respondent per year, we calculated the sum of all responses for cases of symptomatic thoracic endometriosis managed over the course of the respondents’ careers using the lower limit of each range and divided it by the sum of years spent managing patients with endometriosis using the upper limit of each range. This provided an estimation of the minimum number of cases of thoracic endometriosis requiring management per respondent per year. To estimate the maximum number of cases per respondent per year, we calculated the sum of all responses for cases of symptomatic thoracic endometriosis managed over the course of the respondents’ careers using the upper limit of each range and divided it by the sum of years spent managing patients with endometriosis using the lower limit of each range. This provided an estimation of the maximum number of cases of thoracic endometriosis requiring management per respondent per year.

## Results

We sent email questionnaires to all members of the BSGE (635 senior members). We received 67 responses (response rate 11%). We were unable to assess how many participants opened the survey but chose not to participate. Explanations for not participating were not sought.

Two data coders (MH and LB) evaluated the results using Microsoft excel version 16.41 (trade name). The data was evaluated, and the themes explored in the narrative below.

Respondent Demographics:

- How many years have you spent managing women with endometriosis associated pain?
- How many women per year do you manage (medically or surgically) with endometriosis associated pain?
- In your career, how many women have you managed with pain or other symptoms suggestive of thoracic (diaphragm and above) endometriosis?
- How were the patients with thoracic endometriosis referred to you?

Eighteen respondents (27%) managed fewer than 50 women with endometriosis-associated pain per year, 12 respondents (18%) managed 51 to 100 women with endometriosis-associated pain per year, 11 respondents (16%) managed 101 to 150 women with endometriosis-associated pain per year, nine respondents (13%) managed 151 to 200 women with endometriosis-associated pain per year and 17 respondents (25%) managed over 200 women with endometriosis-associated pain per year.

Fifty-five respondents (85%) had managed such patients for over five years, with 43 respondents (66%) having done so for over ten years.

Cumulatively, this survey drew upon over 800 years of clinician experience managing endometriosis. Forty-seven respondents (70%) recalled managing ten or fewer women with thoracic endometriosis over the course of their career. Of those clinicians who had managed patients with thoracic endometriosis and recalled the referral pathway (n=39), 20 respondents 51% reported that such patients had been referred by general practitioners, three respondents (8%) reported that such patients had been referred by respiratory physicians and one respondent (3%) answered “other” i.e. reported that such patients had been referred by a healthcare professional that was not a general practitioner, respiratory physician or cardiothoracic surgeon. The remainder of the respondents had received referrals from a variety of professionals including general practitioners, respiratory physicians, cardiothoracic surgeons and other health professionals.

Screening:

- In patients with pelvic endometriosis, do you routinely enquire about symptoms suggestive of thoracic endometriosis (such as catamenial chest pain, catamenial haemoptysis or recurrent pneumothorax)?
- During laparoscopy to investigate pelvic pain, do you routinely inspect the diaphragm for endometriosis?

When clinicians were asked whether they routinely enquire about symptoms suggestive of thoracic endometriosis during history taking, three (4%) answered ‘always’, nine (13%) answered ‘most of the time’, 11 (16%) answered ‘some of the time’, 15 (22%) answered ‘a little of the time’ and 29 (43%) answered ‘never’. When clinicians were asked whether, during laparoscopy for pelvic pain, respondents routinely inspect the diaphragm for endometriosis, 46 (69%) answered ‘always’, six (9%) answered ‘most of the time’, three (4%) answered ‘some of the time’, three (4%) answered ‘a little of the time’ and nine (13%) answered ‘never’.

Diagnosis:

- In your career, through direct vision or histological confirmation, how many women have you diagnosed with asymptomatic diaphragmatic endometriosis?
- In your career, through direct vision or histological confirmation, how many women have you diagnosed with symptomatic diaphragmatic endometriosis?
- In your career, through imaging, direct vision or histological confirmation, how many women have you diagnosed with symptomatic endometriosis above the diaphragm?
- In reference to symptomatic endometriosis above the diaphragm, how was this diagnosed?

Forty-five respondents (67%) reported having diagnosed asymptomatic diaphragmatic endometriosis either via direct vision or histological confirmation, 16 of which (24%) had diagnosed over 25 cases. Forty-two respondents (63%) reported having diagnosed symptomatic diaphragmatic endometriosis either via direct vision or histological confirmation, ten of which (15%) had diagnosed over ten cases.

Just over a third (24/66) of respondents had diagnosed symptomatic endometriosis above the diaphragm (i.e. bronchopulmonary or pleural endometriosis). Amongst these clinicians, 21/24 respondents (88%) had diagnosed fewer than five patients, two (8%) had diagnosed 6 to 10, and one (4%) had diagnosed 11 to 15 patients. Of those clinicians who had cared for patients with symptomatic endometriosis above the diaphragm (n=24), two respondents (8%) reported the diagnoses were made with CT alone, seven (29%) reported the diagnoses were made with MRI alone, one respondent (4%) reported the diagnoses were made with bronchoscopy alone, six respondents (25%) reported the diagnoses were made with thoracoscopy alone, and eight respondents (33%) reported the diagnoses were made with a variety of two or more of the above diagnostic modalities.

Management

- In reference to symptomatic diaphragmatic endometriosis, how was this managed?
- If you chose surgery for symptomatic diaphragmatic endometriosis, please indicate which procedure was utilised?
- In reference to symptomatic endometriosis above the diaphragm, how was this managed?
- If you chose surgery for symptomatic endometriosis above the diaphragm, please indicate which procedure was utilised?

Amongst those clinicians who had diagnosed symptomatic diaphragmatic endometriosis (n=42), ten respondents (24%) had managed all patients with surgery followed by hormonal therapies, six respondents (14%) had managed all patients with hormonal therapies alone, five respondents (12%) had managed all patients with surgery alone, and one respondent (2%) had managed all patients conservatively. Twenty respondents (48%) had used a variety of the above therapies to manage symptomatic diaphragmatic endometriosis. Among the 37 clinicians who had treated patients with symptomatic diaphragmatic endometriosis surgically, nine respondents (24%) reported that their patients had surgical ablation performed by a gynaecologist, one respondent (3%) reported their patients had surgical ablation by a non- gynaecologist, six respondents (16%) reported that their patients had surgical excision performed by a gynaecologist and seven respondents (18%) reported their patients had undergone surgical excision by a non-gynaecologist. Fourteen respondents (38%) had cared for patients who had undergone a combination of the aforementioned surgical techniques.

The management of symptomatic endometriosis above the diaphragm (n=24) varied. No clinician reported managing patients conservatively, five respondents (21%) reported managing all such patients with hormonal therapies alone, two respondents (8%) had managed all such patients with surgery alone and 11 respondents (46%) had managed all such patients with surgery followed by hormonal therapies. Five respondents (21%) had used a variety of the above therapies.

A small group of gynaecological clinicians (n=19) reported experience treating patients with symptomatic endometriosis above the diaphragm surgically. Amongst these, 14 respondents (74%) reported that their patients underwent thoracoscopic excision, two respondents (11%) reported that their patients underwent open cardiothoracic surgery, and one respondent (5%) reported bronchoscopic excision. Two respondents (11%) had cared for patients who had undergone a combination of surgical techniques.

Service provision

- Which specialists should be part of the team involved in the care of women with thoracic endometriosis?
- How do you feel symptomatic British women with thoracic (diaphragm and above) endometriosis should be managed?

When clinicians were asked which professionals should be part of the team managing women with thoracic endometriosis, 47 respondents (71%) suggested four or more different healthcare professionals. The commonest response (11 respondents; 17%) was: gynaecological surgeon, upper gastrointestinal surgeon, cardiothoracic surgeon, respiratory physician, specialist cardiothoracic anaesthetist, pain specialist, nurse specialist & physiotherapist.

When asked where patients with thoracic endometriosis should be managed, 36 respondents (56%) patients should be managed at a regional or national centre. Two respondents (3%) felt such patients should be managed at an international centre and only a minority, six respondents (9%), felt such patients could be managed at all BSGE centres.

## Discussion

This is the first study seeking to investigate current practice and establish expert opinion on thoracic endometriosis amongst gynaecological specialists. Our study confirms that UK endometriosis specialists perform poorly at screening for thoracic endometriosis with care distributed amongst many centres employing varied diagnostic and treatment strategies. The majority of respondents believed that patients should be managed at a regional or national centre by a multi-disciplinary team of specialists.

This is the first study to evaluate the practice of endometriosis specialists in managing patients with thoracic endometriosis. Our sample population was comprised of senior clinicians with over 800 years combined experience managing endometriosis patients.

This study is not without its limitations. Surveying clinicians about a rare disease manifestation is inherently susceptible to recall bias. We received 67 (11%) responses from 635 consultant members of the BSGE. While the response rate is low, the sample population were experienced with the majority managing over 100 patients annually for over ten years. There is a risk of responder bias with a higher proportion of those clinicians having had experience of thoracic endometriosis choosing to participate. This may lead to an over estimation in the national statistics. However, this represents a well-qualified group of clinicians able to provide expert insight ensuring a representative sample of clinicians nationally. The design of the questionnaire has methodological limitations when performing a qualitative analysis. There were no quotation options available for participants and minor themes were not explored due to the closed ended nature of the questionnaire. The respondents to the survey will receive feedback via dissemination of this publication through the BSGE.

The timing of clinical symptoms with menstruation is believed to be pathognomic, making a clinical history crucial in the diagnostic process. Screening for symptoms of disease is essential for diagnosis. These form a test that leads clinicians down a cascade of further questions and investigations to rule in or rule out the condition. In a clinic setting only three percent always asked about respiratory symptoms limiting the opportunity for further investigations. Despite an experienced cohort of respondents 36% had never managed a case of thoracic endometriosis. These findings suggest that there may be underdiagnosis from poor screening methods in the clinic setting. The results of this study suggest that gynaecological endoscopists are underperforming in the area of symptom screening.

The surgical technique to treat this disease is complex and often led by cardiothoracic surgeons. Given that endometriosis above the diaphragm is rare without coexistent pelvic endometriosis (Joseph and Sahn, 1996), previous reviews have advocated for a combined thoracoscopic and laparoscopic procedure for such patients(Nezhat et al., 2019). The capacity to adopt such an approach would be difficult without routine symptom screening in a centre offering specialist multidisciplinary services.

A lack of widespread experience of managing thoracic endometriosis amongst endometriosis specialists was highlighted by the variation in diagnostic and treatment modalities used. This reflects the lack of national guidance or an established management pathway for patients with thoracic endometriosis in the UK and internationally. The European Society of Human Reproduction and Embryology guideline on the management of endometriosis were only able to make GRADE D level recommendations broadly addressing all forms of extragenital endometriosis. (Dunselman et al., 2014; Neumann et al., 2015) A guideline for the management of thoracic endometriosis published in 2018 by the Collège National des Gynécologues et Obstétriciens Français in conjunction with Haute Autorité de Santé (Merlot et al., 2018) included ten studies evaluating diagnostic (Alifano et al., 2003; Marshall et al., 2005), or therapeutic interventions (Ceccaroni et al., 2012; Duyos et al., 2014; Leong et al., 2006; Nakashima et al., 2011; Nezhat et al., 1998, Nezhat et al., 2014; Suwatanapongched et al., 2015; Yisa et al., 2004) on 122 women. Two of these were case reports (Nakashima et al., 2011; Yisa et al., 2004); three assessed surgical methods (Ceccaroni et al., 2012; Nakashima et al., 2011; Nezhat et al., 1998, Nezhat et al., 2014); two assessed medical therapies alone (Suwatanapongched et al., 2015; Yisa et al., 2004); and two assessed surgical treatments in conjunction with adjuvant medical therapy (Duyos et al., 2014; Leong et al., 2006). There are no randomised control trials in the management of thoracic endometriosis. The paucity of available evidence-based treatment guidelines in this rare but highly morbid form of disease is reflected in the variation in care which women receive across the UK.

To minimise variation in care and aid the generation of future research centralised care is required. NHS England defines highly specialised services as those providing care for rare or complex diseases provided to a smaller number of patients compared to specialised services, usually no more than 500 patients a year (NHS England, 2016). There are 76 such services currently commissioned in the UK, in which care is provided in a small number of specialised centres with examples including choriocarcinoma service (two national centres), fetal spina bifida service (one national centre) and gender identity service (two national centres) allied to our speciality (NHS England, 2016).

There are an estimated 5000 new cases of severe endometriosis annually in the UK suggesting 5% of presentations (250 patients) would suffer with thoracic disease. We estimated the minimum and maximum number of cases of thoracic endometriosis requiring intervention per respondent per year in our survey. The extrapolation of our data suggests that 27 to 60 cases of symptomatic thoracic endometriosis are managed annually by the respondents to this survey. The survey population represents 11% of all senior endometriosis specialists therefore the estimated number of cases of thoracic endometriosis requiring intervention in the UK annually is likely to be between 245- 545. This is comparable to other rare or complex disorders. These results call for the development of a dedicated referral and treatment pathway for patients with thoracic endometriosis, with the delivery of care in a single specialised centre where clinical experience is concentrated, ensuring treatment is standardised, multidisciplinary, and cost effective.

This study confirms that thoracic endometriosis is poorly screened for amongst clinicians with varied management lacking a common diagnostic or therapeutic pathway in the United Kingdom. It has shown a widespread consensus amongst BSGE members that patients would benefit from multidisciplinary care at a regional or national centre. This underlines the importance of developing a national consensus among all stakeholders on the diagnosis and management of women with thoracic endometriosis within a dedicated multidisciplinary service at a specialised centre.
